# Anti-Inflammatory and Antimicrobial Effects of Estradiol in Bovine Mammary Epithelial Cells during* Staphylococcus aureus* Internalization

**DOI:** 10.1155/2016/6120509

**Published:** 2016-03-01

**Authors:** Ivan Medina-Estrada, Joel E. López-Meza, Alejandra Ochoa-Zarzosa

**Affiliations:** Centro Multidisciplinario de Estudios en Biotecnología, Facultad de Medicina Veterinaria y Zootecnia, Universidad Michoacana de San Nicolás de Hidalgo, Km 9.5 Carretera Morelia-Zinapécuaro, Posta Veterinaria, 58893 Morelia, MICH, Mexico

## Abstract

17*β*-Estradiol (E2), the predominant sexual hormone in females, is associated with the modulation of the innate immune response (IIR), and changes in its levels at parturition are related to intramammary infections, such as mastitis. In bovine mammary epithelial cells (bMECs), E2 regulates differentiation and proliferation, but its immunomodulatory functions have not been explored.* Staphylococcus aureus* is the predominant pathogen causing mastitis, which can persist intracellularly in bMECs. The aim of this work was to analyze whether E2 modulates the IIR of bMECs during* S. aureus* internalization. bMECs treated with E2 (50 pg/mL, 24 h) reduced bacteria internalization (~50%). The host receptors *α*5*β*1 and TLR2 do not participate in this reduction. However, E2 activates ER*α* and modulates the IIR reducing the* S. aureus* induced-mRNA expression of TNF-*α* (~50%) and IL-1*β* (90%). E2 also decreased the secretion of these cytokines as well as IL-6 production; however, in infected bMECs, E2 induced the secretion of IL-1*β*. Furthermore, E2 upregulates the expression of the antimicrobial peptides DEFB1, BNBD5, and psoriasin S100A7 (~5-, 3-, and 6-fold, resp.). In addition, E2 induced the production of antimicrobial compounds in bMEC culture medium, which, together with the modulation of the IIR, could be related to the reduction of* S. aureus* internalization.

## 1. Introduction

Estrogens play crucial roles in the development and maintenance of normal sexual and reproductive function. 17*β*-Estradiol (E2) is the predominant and most potent sexual hormone during the reproductive stage in females [1]. Its main functions are associated with reproduction, although it is also involved with different pathologies, such as cancer, autoimmune diseases, and infectious processes, in which the innate immune response plays a key function [2]. In bovines, during the period around parturition, cows experience an increased susceptibility to inflammatory disorders in the mammary gland and uterus, for example, mastitis [3]. This increased susceptibility has been correlated with the decreased functionality of the components of the innate immune response and with abrupt changes in the levels of sex steroids. Parturition and the onset of lactation comprise extensive alterations in the sex steroid hormones. E2 levels rise abruptly in the last week before parturition, peak in the last 3 days before delivery, and fall rapidly after calving and regain basal values [3, 4]. These changes may be related to the incidence of intramammary infections such as mastitis. Accordingly, Lavon et al. [5] reported that cows with subclinical mastitis (without apparent signs) exhibit low circulating E2 levels. In addition, the immunomodulatory effects of E2 in cows with mastitis are associated with a reduction of neutrophil migration [6].

Most of the genomic actions of estrogens, including E2, are mediated by two estrogen receptors (ERs), ER*α* and ER*β*, and bovine mammary epithelial cells express both [7]. However, there is little information concerning the immunomodulatory effects of E2 on epithelial cells from the bovine mammary gland. It is well known that E2 is required for mammary epithelial cell proliferation and ductal development in the growing animal [7]. Bovine mammary epithelial cells (bMECs) play a relevant role during intramammary infections because they are in intimate contact with the pathogens responsible for mastitis and are a target for intracellular bacteria causing chronic and subclinical infections, such as* Staphylococcus aureus* [8, 9]. This pathogen can persist for long periods of time in cows and is internalized by a zipper-type mechanism depending on the presence of fibronectin-binding proteins (FnBPs) on the bacterial surface, as well as the interaction of fibronectin and the host-cell *α*5*β*1 integrin, in which integrin is taken up together with its ligands [9]. This mechanism is employed during the internalization process of* S. aureus* by bMECs [10]. In addition, bMECs participate in the innate immune response of the bovine mammary gland producing pro- and anti-inflammatory mediators, as well as antimicrobial peptides, nitric oxide, and so forth [11, 12], but the role of E2 on this response has not been explored. Considering the immunomodulatory effects of E2 in different tissues and that one of its targets is the mammary epithelium, the aim of this work was to analyze whether this hormone modulates the innate immune response of bMECs and its implications during* S. aureus* internalization.

## 2. Materials and Methods

### 2.1. Reagents and Antibodies

17*β*-Estradiol was acquired from Sigma, and working solutions were dissolved in 1% ethanol (1–500 pg/mL). For all of the experiments, 1% ethanol (vehicle) was used as a control. The LIVE/DEAD BacLight bacterial viability kit (Thermo Scientific) was obtained from Molecular Probes. The monoclonal blocking antibodies anti-*α*5*β*1 integrin (MAB2514) and anti-TLR2 (TLR2.1) were obtained from Millipore and Abcam, respectively. The anti-phospho-ER*α* (2511S, Ser118) was obtained from Cell Signaling and the anti-ER*β* (517700) was acquired from Life Technologies. The FITC-conjugated secondary antibodies against mouse and rat IgGs were purchased from Invitrogen and Thermo Scientific, respectively.

### 2.2. *Staphylococcus aureus* Strain

The* S. aureus* subsp.* aureus* (ATCC 27543) strain was used. This strain was isolated from a case of bovine clinical mastitis and has the capacity to invade bMECs [13].* S. aureus* were grown at 37°C overnight in Luria-Bertani broth (LB Bioxon), and the CFUs were adjusted by measuring the optical density at 600 nm (OD 0.2 = 9.2 × 10^7^ CFU/mL).

### 2.3. Primary Culture of Bovine Mammary Epithelial Cells (bMECs)

bMECs were isolated from the alveolar tissue of the udders of healthy lactating cows as previously described [13]. Cells from passages 2–8 were used in all of the experiments. The bMECs were cultured in growth medium (GM) that was composed of a DMEM medium/nutrient mixture F12 Ham (DMEM/F12K, Sigma) supplemented with 10% fetal calf serum (Equitech Bio), 10 *μ*g/mL insulin (Sigma), 5 *μ*g/mL hydrocortisone (Sigma), 100 U/mL penicillin, 100 *μ*g/mL streptomycin, and 1 *μ*g/mL amphotericin B (Invitrogen). The cells were grown in a 5% CO_2_ atmosphere at 37°C. To perform the E2 and/or* S. aureus* challenge, polarized monolayers of bMECs (dishes covered with 6–10 *μ*g/cm^2^ rat-tail type I collagen, Sigma) were cultured in serum-free DMEM/F12K without phenol red (Sigma) and antibiotics (incomplete medium) for 24 h, and then they were treated with the hormone and/or infected with the bacteria.

### 2.4. Invasion Assays

For the invasion assays, bMEC polarized monolayers were used (~10,000 cells were cultured onto 96-well flat-bottom dishes (Corning)), which were incubated with different concentrations of E2 (1, 5, 10, 50, 150, 300, and 500 pg/mL) in DMEM/F12K (Sigma) without antibiotics, serum, and phenol red (incomplete medium) for 24 h and then were infected with* S. aureus* (MOI 30 : 1 bacteria per cell). For this, the bMECs were inoculated with bacterial suspensions from a broth of 9.2 × 10^7^ CFU/mL and incubated for 2 h in 5% CO_2_ at 37°C. Then, the cells were washed three times with PBS (pH 7.4) and incubated with incomplete medium that was supplemented with 80 *μ*g/mL gentamicin for 1 h at 37°C to eliminate extracellular bacteria. Finally, the bMEC monolayers were detached with trypsin- (0.05%) EDTA (0.02%) (Sigma) and lysed with 250 *μ*L of sterile distilled water. The bMEC lysates were diluted 100-fold, plated on LB agar in triplicate, and incubated overnight at 37°C. The number of CFUs was determined by the standard colony counting technique. The number of bMECs cultured in each well plate was calculated for each invasion assay using an automated cellular counter (BIO-RAD, TC20). The data are presented as the ratio of the CFU recovered per bMEC.

For the invasion assays in the presence of the blocking antibodies, one hour previous to the addition of* S. aureus* to the bMECs, the blocking antibodies anti-*α*5*β*1 integrin or anti-TLR2 were added separately (10 and 5 *μ*g/mL, resp.) to triplicate wells. Rat IgGs (purified from normal rat serum with protein A-sepharose beads (Sigma)) or mouse IgGs (purified from normal mouse serum and acquired from Pierce) were used as the negative controls. The invasion assays were performed using gentamicin protection assays as previously described [13].

### 2.5. bMEC Viability and* S. aureus* Growth Assays

To determine the effect of E2 on bMEC viability, 10,000 cells were incubated with different concentrations of the hormone in incomplete medium for 24 h at 37°C in 96-well plates. Then, 10 *μ*L of a 5 mg/mL of 3-(4,5-dimethyl-2-thiazolyl)-2,5-diphenyl-2H-tetrazolium bromide (MTT, Sigma) solution in PBS was added to each well and incubated for 4 h at 37°C. Finally, 100 *μ*L of acid isopropanol (95% isopropanol and 5% 1 N HCl) were added to dissolve the formazan crystals. The optical density was measured with a microplate reader (BIO-RAD) at 595 nm.

The bMEC viability was also tested using the trypan blue exclusion assay and the cells were counted in an automated cell counter (BIO-RAD, TC20).

To analyze the effect of E2 on* S. aureus* growth, 9.2 × 10^7^ CFU/mL were cultured at 37°C in LB broth. The bacterial suspensions were treated with different concentrations of the hormone (1, 5, 10, 50, 150, 300, and 500 pg/mL) and growth was monitored turbidimetrically (600 nm) over 24 h (measuring the absorbance at 2, 4, 6, 8, 12, and 24 h). To evaluate the* S. aureus* viability in the presence of E2, bacteria were grown (as previously described) in LB broth and were treated with E2 for 24 h at 37°C overnight. Later, the bacteria were plated on LB agar in triplicate and were incubated overnight at 37°C. The number of CFUs was determined by standard colony counting.

Bacterial viability was also analyzed in the supernatants of the infection assays. To do this, the bMECs were treated with E2 and infected with* S. aureus* as described above. After 2 h of infection, the media were recovered and the bacteria pellet was obtained by centrifugation at 10,000 rpm for 10 min at 4°C and was washed three times with PBS. The* S. aureus* viability was measured using a LIVE/DEAD BacLight Bacterial viability kit (Thermo Scientific). The pellet was incubated with equal volumes (1.5 mL) of component A (SYTO 9 dye, live indicator) and component B (propidium iodide, dead indicator) at room temperature in the dark for 15 min. After staining the bacteria, the pellet was washed three times with PBS. The fluorescent signals of 10,000 events were measured and evaluated using a BD Accuri*™* C6 cytometer. In addition,* S. aureus* growth was also analyzed using the E2-treated bMEC conditioned media. To do this, the bacteria were grown at 37°C overnight, and a* S. aureus* suspension containing 9.2 × 10^7^ CFU/mL (OD 0.2 at 600 nm) was incubated at 37°C for 2 h with the conditioned media. Then, the bacteria were plated on LB agar in triplicate and were incubated overnight at 37°C. The number of CFUs was determined as described previously.

### 2.6. Analysis of Receptors by Flow Cytometry

The bMEC polarized monolayers were cultured in 24-well dishes (Corning) and were treated with E2 (50 pg/mL) for 24 h and/or* S. aureus* as described above. This concentration was selected considering the highest inhibitory effect on bacterial internalization into bMECs. After the treatment, the cells were washed three times with PBS, detached with trypsin- (0.05%) EDTA (0.02%) (Sigma), centrifuged at 2,500 rpm for 10 min at 4°C, and washed with PBS. The bMEC pellet was blocked with normal goat serum (5% in PBS, Pierce) for 30 min at 4°C with shaking, and then the cells were centrifuged and the pellet was incubated with the primary antibodies anti-TLR2 or anti-integrin separately. To analyze the presence of the ERs (ER*α* or ER*β*), nuclei from the bMECs were obtained using the ProteoJet kit (Fermentas), and the procedure continued as described above. To measure the TLR2 membrane abundance (MA) or the nuclear presence of the ERs, the cells were incubated with the antibody at a dilution of 1 : 50 (PBS containing BSA 0.1%) for 1 h at 4°C with shaking. For the determination of integrin MA, invasion assays were performed using different infection times: 30, 60, and 120 min in control bMECs or E2-treated (50 pg/mL, during 24 h) bMECs. The invasion assay was completed as described previously. The cells were incubated with the anti-*α*5*β*1 integrin at a concentration of 10 *μ*g/mL for 2 h at 4°C with shaking. In all cases, after the primary antibody incubation, the bMECs were washed three times with PBS and incubated with the respective secondary antibody (diluted 1 : 50) (FITC-conjugated anti-mouse IgGs for TLR2 and ER*α* and FITC-conjugated anti-rat IgGs for ER*β* and *α*5*β*1 integrin) for 1 h at 4°C with shaking in the dark. The pellet was recovered by centrifugation, washed, and fixed with paraformaldehyde (4%) for 10 min at 4°C and finally washed three times with PBS. The cells were then suspended in 100 *μ*L of PBS. The fluorescent signals of 10,000 events were measured and evaluated using the BD Accuri C6 cytometer.

### 2.7. Role of MAP Kinases in* S. aureus* Internalization into bMECs

The polarized bMEC monolayers were cultivated in 96-well flat-bottom plates that were coated (Corning-Costar) with 6–10 *μ*g/cm^2^ rat-tail type I collagen (Sigma). The bMECs were incubated with E2 (50 pg/mL for 24 h). Prior to the invasion assay (30 min, 1 or 2 h), pharmacological inhibitors of p38 (5 *μ*M, SB203580), JNK (20 *μ*M, SP600125), or ERK1/2 (2.5 *μ*M, U0126) were added separately to the bMECs. The infection assays were performed with the gentamicin protection assays as described above. The cells lysates were plated on LB agar in triplicate and incubated overnight at 37°C. The CFU numbers were determined with the standard colony counting technique or* S. aureus* viability was measured using the LIVE/DEAD BacLight Bacterial viability kit as described above. 0.1% DMSO (vehicle) was used as a control.

To evaluate the MAP kinase activation levels by flow cytometry, the bMECs were treated with E2 (50 pg/mL),* S. aureus,* or both, and the samples (30 *μ*g of protein) were prepared according to the manufacturer's protocol for adherent cells (Becton Dickinson, Germany). pp38 (T180/Y182), pJNK1/2 (T183/185), and pERK1/2 (T202/Y204) were quantitatively determined using antibodies from a Flex Set Cytometric Bead Array (Becton Dickinson) according to the manufacturer's protocol. The flow cytometric analyses were performed using the BD Accuri C6 and the CBA analysis FCAP software (Becton Dickinson). A total of 3,000 events were acquired following the supplied protocol. The minimum detection levels for each phosphoprotein were 0.38 U/mL for pJNK and 0.64 U/mL for pp38 and pERK.

### 2.8. RNA Isolation and RT-qPCR Analysis

Monolayers of bMECs were cultured in 6-well dishes (~5 × 10^5^) and then were incubated with 50 pg/mL of E2 (24 h) and/or* S. aureus* (MOI 30 : 1) for 2 h, as described above. The RNA was extracted with Trizol reagent (Invitrogen) according to the manufacturer's instructions. DNA contaminations were removed from RNA samples with DNase I treatment (Invitrogen). The RNA was used to synthesize cDNA as described previously [10]. The RT-qPCR assay was performed using the comparative Ct method (ΔΔCt) with a StepOne Plus Real-Time PCR System (Applied Biosystems) according to the manufacturer's instructions. The reactions were carried out with a SYBR Green PCR Master Mix (Applied Biosystems). Glyceraldehyde-3-phosphate dehydrogenase (GAPDH) was used as the internal control. The specific primer pairs were obtained from Invitrogen; their sequences and PCR conditions used to amplify the different bovine mRNAs are detailed in Table 1.

### 2.9. Enzyme-Linked Immunosorbent Assays (ELISA)

For the measurement of the TNF-*α*, IL-1*β*, IL-6, and bovine *β*-defensin 1 (DEFB1) concentrations in the medium, the conditioned media from bMECs treated with E2 (50 pg/mL) and/or* S. aureus* were collected. The concentrations of TNF-*α* were measured using the Duoset ELISA development kit (R&D Systems) to quantify the bovine TNF-*α* levels according to the manufacturer's instructions, and the concentrations of IL-1*β* and IL-6 were assessed using the bovine IL-1*β* and IL-6 screening kits, respectively (Thermo Scientific). The measurement of bovine DEFB1 in bMECs culture medium was analyzed using the ELISA kit for DEFB1 (MyBiosource).

### 2.10. Nitric Oxide Production Assay

The nitric oxide (NO) secreted by bMECs into culture medium was evaluated by measuring the nitrite concentration (NO^2−^) in cell-free media using the Griess reaction [16]. After the invasion assays, the medium was filtered through 0.22 *μ*M membranes (Millipore) to eliminate bacteria.

### 2.11. Data Analysis

The data were obtained from three independent experiments, each of which was performed in triplicate (except for the ELISA of DEFB1, which was performed in duplicate) and compared by analysis of variance (ANOVA). The results are reported as the means ± the standard errors (SE) and the significance level was set at *P* < 0.05, except for RT-qPCR analysis where standard deviations are shown and fold-change values greater than 2 or less than 0.5 were considered as significantly differentially expressed mRNAs according to Morey et al. [17]. The results from internalization assays, gene expression analyses, and receptor membrane abundance experiments are shown normalized to the cells treated with vehicle (1% ethanol).

## 3. Results

### 3.1. Estradiol Does Not Affect Bacterial Growth and bMEC Viability

**Figure 1:**
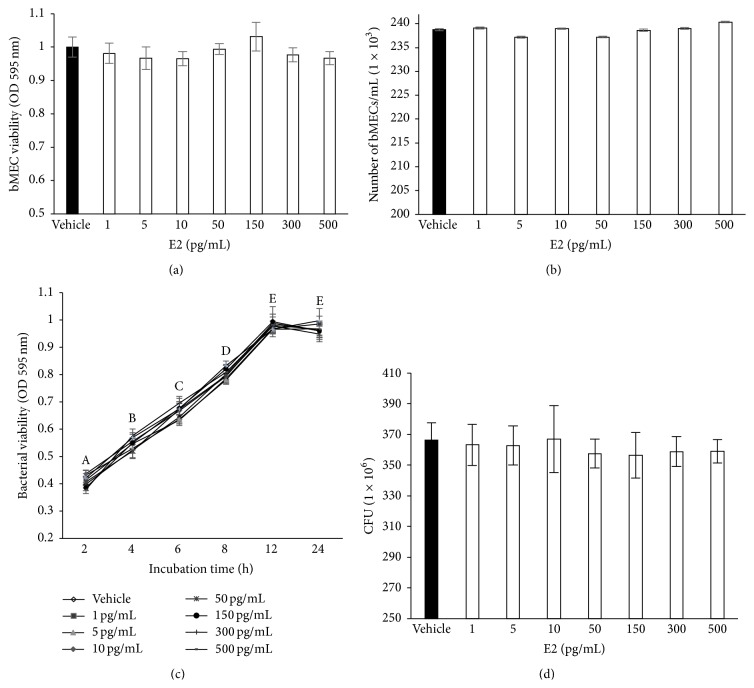
Bovine mammary epithelial cell viability and* S. aureus* growth in the presence of E2. (a) bMECs were cultured with E2 and viability was determined by MTT assay at 24 h. (b) bMECs were cultured with the hormone and viability was calculated by a trypan blue exclusion assay. The number of viable bMECs/mL is shown. In all cases, concentrations evaluated were 1, 5, 10, 50, 150, 300, and 500 pg/mL. (c)* S. aureus* was cultured in the presence of E2 at different times during 24 h and bacteria viability was analyzed by MTT assay. (d) Bacterial growth was determined counting the CFUs of* S. aureus* treated with different concentrations of E2 during 24 h. Each point in (a), (b), (c), and (d) shows the mean of triplicates ± SE of three independent experiments. The vehicle corresponds to bMECs treated with 1% ethanol. Different letters indicate significant changes among the different incubation times (*P* < 0.05).

To evaluate the effect of E2 on* S. aureus* growth and bMEC viability, we incubated bacteria or bMECs in the presence of different concentrations of this hormone (1–500 pg/mL). Bacterial viability was analyzed by counting the CFUs and by the MTT assay. The results showed that E2 did not have effect on bMEC viability after 24 h of culture (Figures 1(a) and 1(b)), which was determined by a trypan blue exclusion analysis and the MTT assays. In the same way, we showed that E2 did not affect the bacterial viability and growth (Figures 1(c) and 1(d)).

### 3.2. Estradiol Reduces* S. aureus* Internalization into the bMECs

**Figure 2:**
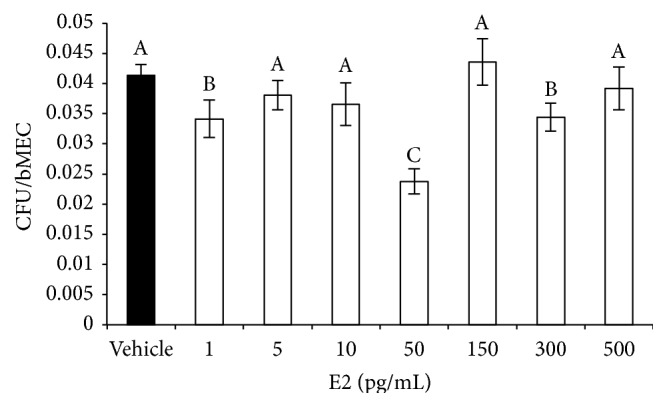
E2 decreases* S. aureus* internalization into bMECs. bMECs were treated with the concentrations indicated of E2 (24 h), and then were challenged with* S. aureus* during 2 h. Data are showed as the percentage of CFU recovered after bMEC lysis. Values were determined considering the control (bMECs cultured with the vehicle 1% ethanol) as 100% internalization. Each bar shows the mean of triplicates ± SE of three independent experiments. Different letters indicate significant changes among treatments (*P* < 0.05).

To determine the effect of E2 on* S. aureus* internalization into the bMECs, we carried out gentamicin protection assays. To achieve this, bMECs were treated with different concentrations of the hormone (1–500 pg/mL) 24 h before the challenge with the bacteria. According to the number of CFUs recovered, a concentration of 50 pg/mL of E2 significantly inhibited* S. aureus* internalization into the bMECs by 50% (Figure 2). The concentrations of 1, 10, and 300 pg/mL also reduced internalization by 20%. The other concentrations did not affect the internalization rate of* S. aureus* into the bMECs. Considering these results, in the following experiments related to the regulation of the innate immune response of bMECs by E2, we analyzed only the concentration of 50 pg/mL.

### 3.3. *α*5*β*1 Integrin Membrane Abundance Is Not Required for the Internalization of* S. aureus* Inhibited by E2

**Figure 3:**
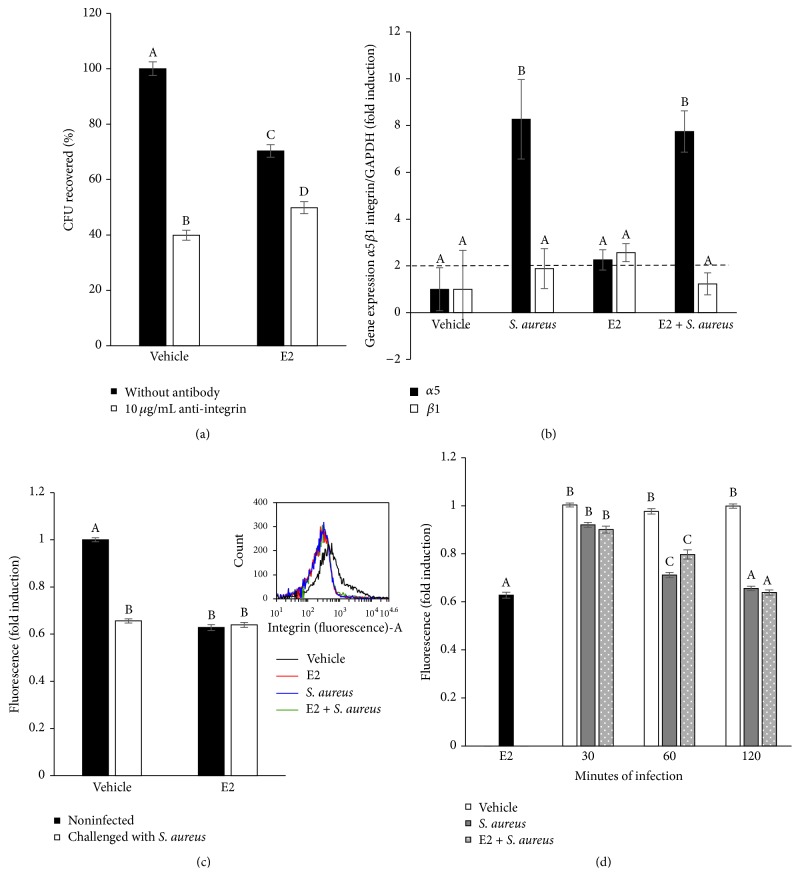
Role of *α*5*β*1 integrin during the internalization of* S. aureus* into bMECs inhibited with E2. (a) Primary cultures of bMECs treated for 24 h with E2 (50 pg/mL) were incubated with 10 *μ*g/mL of a specific blocking antibody against *α*5*β*1 integrin for 1 h and then challenged with* S. aureus* (MOI 30 : 1 bacteria per cell). The number of internalized bacteria is represented by the ratio of CFU/bMEC recovered after lysis of bMECs. (b) The *α*5*β*1 mRNA expression was analyzed by RT-qPCR and GAPDH was used as endogenous gene in all conditions. Fold-change values greater than 2 or less than 0.5 were considered as significant differentially expressed mRNAs. Each bar shows the mean of triplicates ± SD of three independent experiments. (c) The relative fluorescence intensities of *α*5*β*1 membrane abundance in bMECs treated with E2 and infected with* S. aureus* are shown. Fluorescence intensity was estimated from 10,000 events. An inserted histogram plot that shows *α*5*β*1 staining data in vehicle-treated bMECs (black line), cells that were challenged with* S. aureus* (blue line), cells that were stimulated with E2 (red line), and E2-treated bMECs infected with* S. aureus* (green line). The cells were fixed and stained extracellularly with an anti-*α*5*β*1 antibody overnight and analyzed by flow cytometry. (d) The abundance of *α*5*β*1 integrin in the bMEC membrane determined as the mean of the fluorescence of challenged bMECs at different times of infection with the same MOI (30 : 1 bacteria per cell). A total of 10,000 events were measured. Different letters indicate significant changes among treatments (*P* < 0.05), except for (b), where different letters above the bars indicate significant changes among the four treatments within the same gene evaluated (*P* < 0.05). Vehicle: 1% ethanol.

Because *α*5*β*1 integrin is one of the main extracellular receptors of epithelial cells employed during* S. aureus* internalization, we wondered whether the specific functional blocking of *α*5*β*1 integrin would modify the number of bacteria internalized into the bMECs. To test this, primary cultures of bMECs treated for 24 h with E2 were incubated with a specific antibody against *α*5*β*1 integrin (Figure 3(a)). E2 treatment (50 pg/mL) reduced the number of CFUs internalized into the bMECs, but the blockade of *α*5*β*1 integrin slightly reduced this number (~20%). In addition, the bMECs treated with vehicle reduced ~60% of the* S. aureus* internalization when *α*5*β*1 integrin was blocked. This finding suggests that, during the internalization of* S. aureus* into the E2-treated bMECs, integrin *α*5*β*1 does not participate.

To determine whether this effect is correlated with *α*5*β*1 integrin gene expression, we evaluated the gene expression of both subunits by RT-qPCR.* S. aureus* induced the expression of the *α*5 subunit (~8-fold), and E2 upregulated the expression of both subunits. However, the effect of both treatments was similar to the effect of* S. aureus* (Figure 3(b)). To analyze whether this effect is correlated with the *α*5*β*1 integrin MA in the bMECs, we performed a flow cytometry analysis. The mean fluorescence intensity was used as an indicator of the level of integrin expression per cell in the population. This analysis is shown in Figure 3(c). E2 significantly reduces the level of *α*5*β*1 integrin MA (~40%); however, after 2 h of challenge with* S. aureus*, the level of *α*5*β*1 integrin MA was reduced in a similar manner as that of the E2-treated cells. A similar effect was also observed when the E2-treated bMECs were infected, indicating that integrin is taken up together with bacteria. Typical population profiles from the flow cytometry analysis are also shown in Figure 3(c). We additionally evaluated the level of *α*5*β*1 integrin MA at different times of challenge with* S. aureus* in the E2-treated bMECs. The results from this evaluation are shown in Figure 3(d). The presence of *α*5*β*1 integrin at the membrane of the bMECs was reduced starting from 30 min of* S. aureus* challenge, showing a similar behavior both in the control and E2-treated cells. Despite the data showing that E2 reduces integrin MA (24 h treatment), these results suggest that bacteria are able to induce integrin trafficking. Thus, *α*5*β*1 integrin MA does not contribute to the reduction in internalization detected in the E2-treated bMECs (Figure 2).

### 3.4. Regulation of TLR2 Expression by E2 in the bMECs

**Figure 4:**
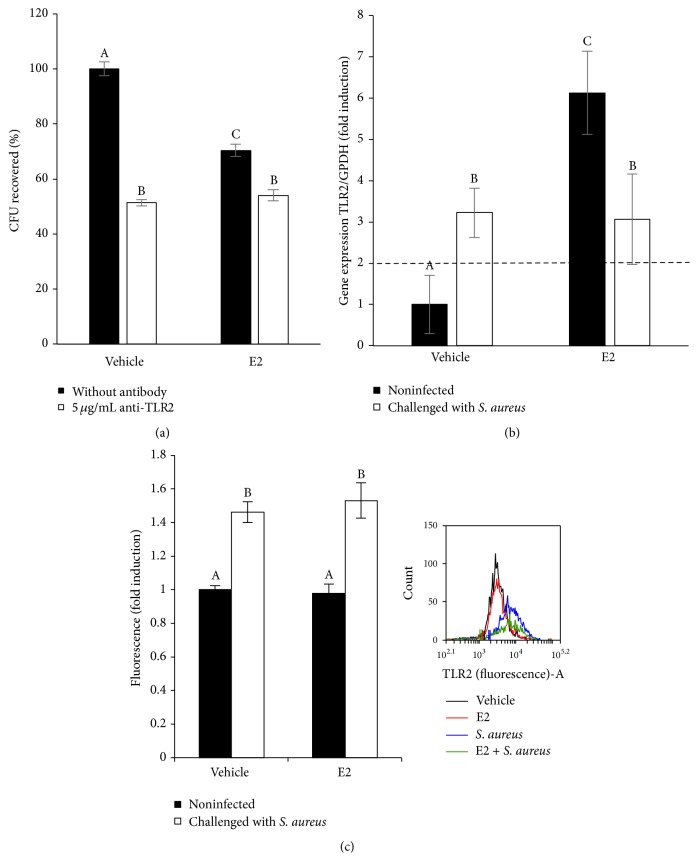
Participation of TLR2 in the internalization of* S. aureus* by bMECs inhibited with E2. (a) Primary cultures of bMECs treated for 24 h with E2 (50 pg/mL) were incubated with 5 *μ*g/mL of a specific blocking antibody against TLR2 for 1 h and then challenged with* S. aureus* (MOI 30 : 1 bacteria per cell). The number of internalized bacteria is represented by the ratio of CFU/bMEC recovered after lysis of bMECs. (b) The TLR2 mRNA expression was analyzed by RT-qPCR and GAPDH was used as endogenous gene in all conditions. Fold-change values greater than 2 or less than 0.5 were considered as significant differentially expressed mRNAs. Each bar shows the mean of triplicates ± SD of three independent experiments. (c) The relative fluorescence intensities of TLR2 membrane abundance in bMECs treated with E2 and infected with* S. aureus* are shown. Fluorescence intensity was estimated from 10,000 events. An inserted histogram plot that shows TLR2 staining data in vehicle-treated bMECs (black line), cells that were challenged with* S. aureus* (blue line), cells that were stimulated with E2 (red line), and E2-treated bMECs infected with* S. aureus* (green line). The cells were fixed and stained extracellularly with an anti-TLR2 antibody overnight and analyzed with flow cytometry. Different letters indicate significant changes among treatments (*P* < 0.05). Vehicle: 1% ethanol.

To explore whether TLR2 participates in the reduction of* S. aureus* internalization into the bMECs induced by E2, we investigated whether the blockade of this receptor with a specific antibody would modify bacterial endocytosis. As shown in Figure 4(a), the blockade of TLR2 results in a significant reduction in the amount of* S. aureus* internalized (~50%); however, a slight reduction was detected in the E2-treated cells (~15%). Surprisingly, E2 induces TLR2 gene expression (~6-fold) (Figure 4(b)). As we previously reported [10],* S. aureus* induces TLR2 mRNA expression (~3-fold); however, the effect of both treatments was similar to the effect of* S. aureus* alone. The analysis of the mean fluorescence intensity (Figure 4(c)) shows that only* S. aureus* (~1.4-fold) increased the TLR2 MA, but there is no difference in the TLR2 MA in the presence of E2 and bacteria compared with that obtained with infection alone. Thus, TLR2 could not be involved in* S. aureus* internalization, and it is probably inactivated in E2-treated bMECs.

### 3.5. Activation of TLR2 Signaling Pathways by E2 and* S. aureus*

**Figure 5:**
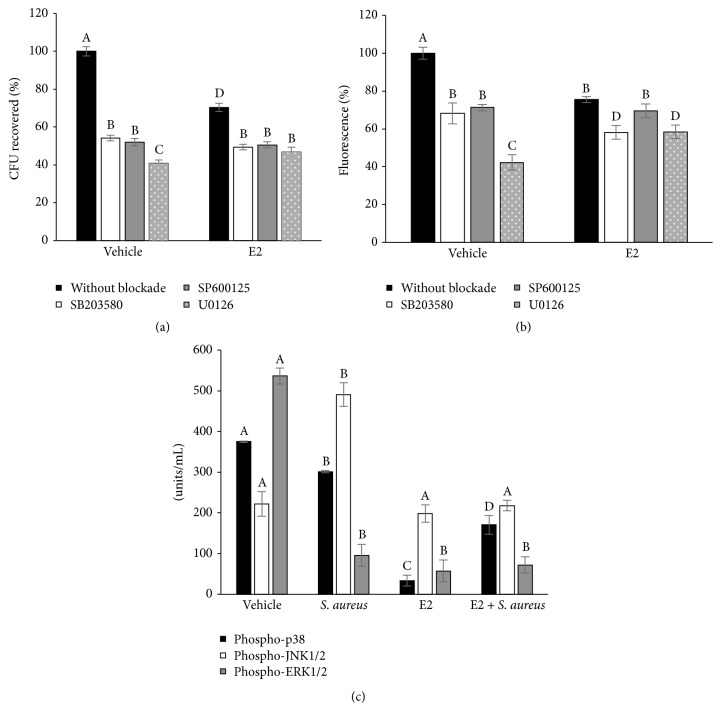
Role of MAPKs p38, JNK, and ERK1/2 in E2-treated bMECs during* S. aureus* internalization. (a) Participation of MAPKs in* S. aureus* internalization determined by CFU units. bMECs were incubated (30 min, 1 or 2 h) with pharmacological inhibitors of p38 (5 *μ*M, SB203580), JNK (20 *μ*M, SP600125), or ERK1/2 (2.5 *μ*M, U0126) prior to infection with* S. aureus* (2 h). Values were determined considering the control (bMECs cultured with the vehicle 1% ethanol) as 100% internalization. (b) The same procedure was used to determine* S. aureus* internalization by flow cytometry using the LIVE/DEAD BacLight Bacterial viability kit. Fluorescence intensity was estimated from 10,000 events. (c) Phosphorylation was measured in bMECs that were treated with 50 pg/mL E2 and/or challenged with* S. aureus* by flow cytometry. The phosphorylated MAPK concentrations (U/mL) are represented: (a) pp38, (b) pJNK, and (c) pERK1/2. SB203580: p38 inhibitor. SP600125: JNK1/2 inhibitor. U0126: ERK1/2 inhibitor. According to manufacturer's protocol 300 events were obtained. In (a) and (b) different letters indicate significant changes among treatments (*P* < 0.05), and in (c) different letters above the bars indicate significant changes among the four treatments within the same MAPK evaluated (*P* < 0.05). Vehicle: 1% ethanol.

Next, we evaluated whether E2 (50 pg/mL) induces the activation of MAPKs (p38, JNK, or ERK1/2), which are activated by TLR2. Initially, we investigated whether these kinases participate in* S. aureus* internalization into bMECs using pharmacological inhibitors prior to bacterial invasion. bMECs that were incubated (for 30 min or 1 or 2 h) with pharmacological inhibitors of p38 (5 *μ*M, SB203580), JNK (20 *μ*M, SP600125), or ERK1/2 (2.5 *μ*M, U0126) showed a considerable reduction in* S. aureus* internalization (~50–60% reduction), indicating that these kinases are involved in this process (Figure 5(a)).* S. aureus* internalization was also analyzed by CFU counting and flow cytometry, which obtained similar results (Figure 5(b)). Interestingly, these kinases do not participate in the reduction of bacterial internalization induced by E2 because in the presence of inhibitors this reduction persists.

Furthermore, we evaluated p38, JNK, and ERK1/2 phosphorylation to relate their activation states with the E2-mediated reduction of* S. aureus* internalization into bMECs. When the bMECs were* S. aureus*-challenged (2 h), the basal activation of p38 was not modified, phosphorylated JNK1/2 was augmented, and ERK1/2 activation was reduced (Figure 5(c)), as we previously reported [18]. The vehicle employed for the MAPK dilutions was 0.1% DMSO, which did not have an effect on internalization assays (data not showed), as was previously reported [18]. Interestingly, the bMECs that were treated with E2 demonstrated reduced p38 phosphorylation (~5-fold), and the ERK1/2 activation levels were also reduced by ~1-fold, but JNK1/2 remained unchanged in relation to the control cells. When the E2-treated cells were* S. aureus*-challenged, the p38 activation level was augmented; however, the JNK1/2 and ERK1/2 activation levels were essentially not changed. The MAPK results indicated that E2, prior to bacterial invasion, inhibits the bMEC inflammatory response via p38 and ERK1/2 inactivation.

### 3.6. E2 Induces the Activation of ER*α* in bMECs

**Figure 6:**
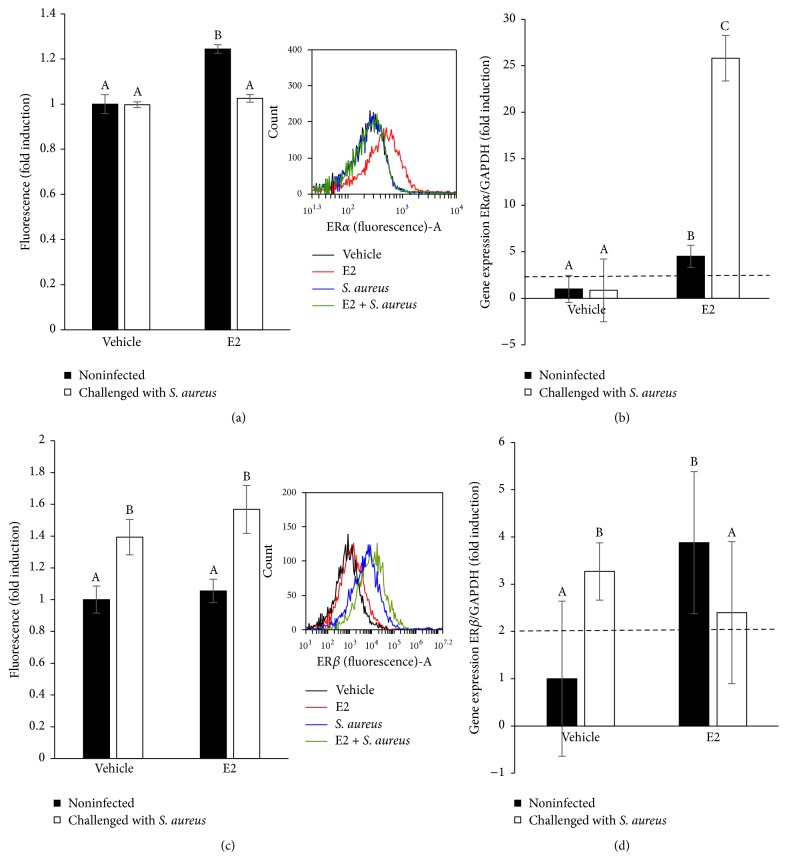
Participation of ERs in E2-treated bMECs. (a) The relative fluorescence intensities of ER*α* activation in bMECs treated with E2 and infected with* S. aureus* are shown. Fluorescence intensity was estimated from 10,000 events. An inserted histogram plot that shows ER*α* staining data in vehicle-treated bMECs (black line), cells that were challenged with* S. aureus* (blue line), cells that were stimulated with E2 (red line), and E2-treated bMECs infected with* S. aureus* (green line). The bMEC nuclei were fixed and stained with an anti-pER*α* antibody overnight and analyzed with flow cytometry. (b) The ER*α* mRNA expression was analyzed by RT-qPCR and GAPDH was used as endogenous gene in all conditions. Fold-change values greater than 2 or less than 0.5 were considered as significant differentially expressed mRNAs. Each bar shows the mean of triplicates ± SD of three independent experiments. (c) The relative fluorescence intensities of ER*β* activation in bMECs treated with E2 and infected with* S. aureus* are shown. Fluorescence intensity was estimated from 10,000 events. An inserted histogram plot that shows ER*β* staining data in vehicle-treated bMECs (black line), cells that were challenged with* S. aureus* (blue line), cells that were stimulated with E2 (red line), and E2-treated bMECs infected with* S. aureus* (green line). The bMEC nuclei were fixed and stained with an anti-ER*β* antibody overnight and analyzed with flow cytometry. (d) The ER*β* mRNA expression was analyzed by RT-qPCR and GAPDH was used as endogenous gene in all conditions. Fold-change values greater than 2 or less than 0.5 were considered as significant differentially expressed mRNAs. Each bar shows the mean of triplicates ± SD of three independent experiments. Different letters indicate significant changes among treatments (*P* < 0.05). Vehicle: 1% ethanol.

Estrogens act through the estrogen receptors ER*α* and ER*β*, which belong to the nuclear receptor superfamily of transcription factors. To determine whether these receptors are activated in bMECs treated with E2 and/or infected with* S. aureus*, we performed a flow cytometry analysis. We used an anti-ER*α* antibody, which recognizes its phosphorylated isoform, as an indicator of its activation. To perform these analyses, we isolated bMEC nuclei. In Figure 6(a), we show that E2 slightly induces the activation of ER*α* (~20%), which was returned to basal levels during* S. aureus* infection. Regarding the expression of ER*α* mRNA, we observed that E2 also induces the expression of its gene (~5-fold), but after infection this induction is higher (~25-fold) (Figure 6(b)).

With regard to ER*β* activation, we performed flow cytometry analysis on the bMEC nuclei. In Figure 6(c), we show that only the bMECs infected with* S. aureus* induce the activation of this receptor in both vehicle- or E2-treated cells. However, E2 treatment does not induce ER*β* activation. In addition, the expression of ER*β* mRNA was induced both in the E2-treated and the* S. aureus*-challenged bMECs (~3.5-fold) (Figure 6(d)). The infection of the bMECs treated with the hormone maintained the upregulated levels of ER*β* (~2.5-fold). According to these results, we hypothesized that 50 pg/mL E2 activates the bMECs via ER*α*.

### 3.7. E2 Maintains an Anti-Inflammatory Profile in the bMECs

**Figure 7:**
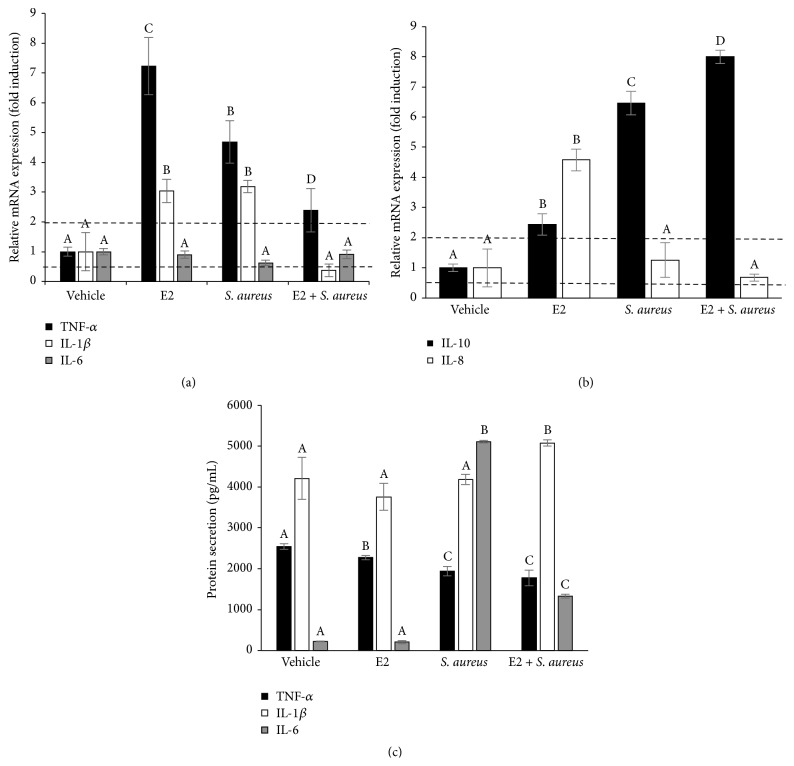
Expression of pro- and anti-inflammatory genes in bMECs treated with E2 and infected with* S. aureus*. (a) RT-qPCR analysis showing the mRNA expression levels of TNF-*α*, IL-1*β*, and IL-6. The bMECs were treated with 0.5 pg/mL of E2 for 24 h and then challenged with* S. aureus* for 2 h. (b) RT-qPCR analysis showing the mRNA expression levels of IL-10 and IL-8. The bMECs were treated with 50 pg/mL of E2 for 24 h and then challenged with* S. aureus* for 2 h. The data are presented as the ratio of the target gene expression compared with the expression level of GAPDH. Each bar shows the mean of triplicates ± SD of three independent experiments. Fold-change values greater than 2 or less than 0.5 were considered as significant differentially expressed mRNAs. (c) Concentrations of TNF-*α*, IL-1*β*, and IL-6 in culture medium of bMECs treated with 50 pg/mL of E2 for 24 h and then challenged with* S. aureus* for 2 h. The protein concentrations were determined by ELISA. Different letters above the bars indicate significant changes among the four treatments within the same gene or cytokine evaluated (*P* < 0.05). Vehicle: 1% ethanol.

Next, we explored whether E2 modified the inflammatory response of the bMECs before and during* S. aureus* infection. We focused on (i) proinflammatory cytokines, such as TNF-*α*, IL-1*β*, and IL-6; (ii) an anti-inflammatory cytokine (IL-10); and (iii) a chemokine, IL-8 (Figure 7). Regarding the proinflammatory cytokines, we performed RT-qPCR for mRNA expression and ELISAs for the evaluation of cytokine secretion. The bMECs treated with E2 (50 pg/mL, 24 h) significantly increased TNF-*α* and IL-1*β* mRNA expression (~7- and 3-fold, resp.) (Figure 7(a)).* S. aureus* infection alone also induced the expression of both cytokines (~5-fold for TNF-*α* and ~3-fold for IL-1*β*). In the presence of* S. aureus*, the E2-treated bMECs reduced the expression of TNF-*α* and IL-1*β* mRNAs. The expression of IL-6 remained unchanged in all of the treatments. Interestingly, in the E2-treated cells the secretion of these proinflammatory cytokines was diminished in relation to bMECs treated with vehicle (Figure 7(c)). Furthermore, 50 pg/mL of E2 also reduced the secretion of TNF-*α* and IL-6 in the infected bMECs. Only the secretion of IL-1*β* was increased when the E2-treated cells were infected with* S. aureus*. In Figure 7(b), we show that E2 also induces the expression of IL-10 and IL-8 mRNAs (~2.5 and 4.5-fold, resp.). The infection induced the expression of IL-10 but together with the hormone increased its expression (~8-fold). The expression of IL-8 was not modified in the presence of infection both in the vehicle- and E2-treated cells. Altogether, these results point to the anti-inflammatory effects of E2 on the bMECs infected with* S. aureus*.

### 3.8. Effects of E2 on the Expression and Secretion of Antimicrobial Molecules in the bMECs

**Figure 8:**
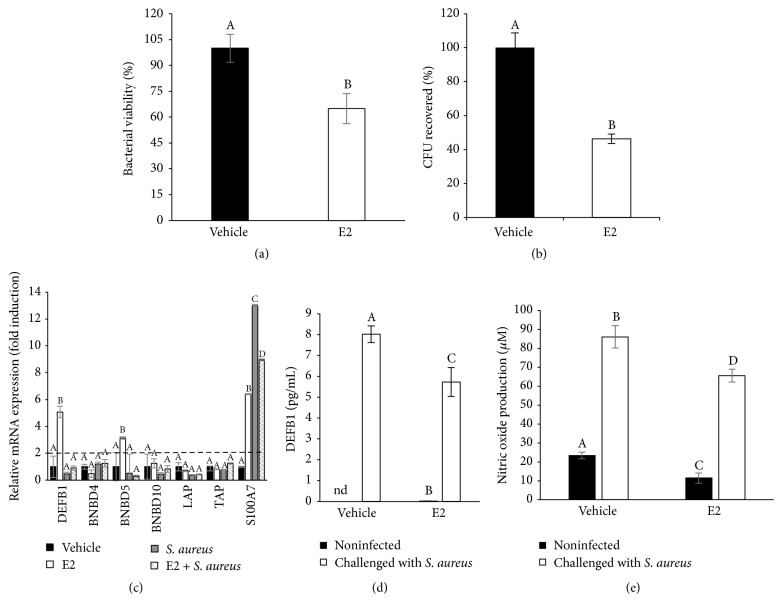
Expression and production of antimicrobial molecules in bMECs treated with E2 and infected with* S. aureus.* (a)* S. aureus* viability from supernatants of infection assays performed in the absence of gentamicin. Noninternalized bacteria viability was analyzed by flow cytometry using the LIVE/DEAD BacLight Bacterial viability kit. Fluorescence intensity was estimated from 10,000 events. Values were determined considering the effect of control (bMECs cultured with the vehicle 1% ethanol) as 100% of viability. (b) Effect of conditioned medium from bMECs treated with vehicle (1% ethanol) or E2 (50 pg/mL) for 24 h on* S. aureus* viability. Bacteria were incubated 2 h with conditioned media and viability was analyzed by flow cytometry using the LIVE/DEAD BacLight Bacterial viability kit. Fluorescence intensity was estimated from 10,000 events. Values were determined considering the effect of control-conditioned medium (bMECs cultured with the vehicle 1% ethanol) as 100% of viability. (c) RT-qPCR analysis showing the mRNA expression levels of DEFB1, BNBD4, BNBD5, BNBD10, LAP, TAP, and S100A7. The bMECs were treated with 50 pg/mL of E2 for 24 h and then challenged with* S. aureus* for 2 h. The data are presented as the ratio of the target gene expression compared with the expression level of GAPDH. Each bar shows the mean of triplicates ± SD of three independent experiments. Fold-change values greater than 2 or less than 0.5 were considered as significant differentially expressed mRNAs. (d) Concentration of DEFB1 in culture medium of bMECs treated with 50 pg/mL of E2 for 24 h and then challenged with* S. aureus* for 2 h. The protein concentrations were determined by ELISA. Each bar shows the mean of duplicates ± SD from different experiments, nd: nondetermined. (e) Nitric oxide production in bMECs treated with 50 pg/mL of E2 for 24 h and then challenged with* S. aureus* for 2 h, measured as NO_2_ concentration in culture medium. Each bar shows the mean of triplicates ± SE of three independent experiments. Different letters indicate significant changes among treatments (*P* < 0.05), except for (c), where different letters above the bars indicate significant changes among the four treatments within the same gene evaluated (*P* < 0.05). Vehicle: 1% ethanol.

To explore if some antimicrobial mediators are being regulated by E2, which could explain the reduction in* S. aureus* internalization, we measured the viability of* S. aureus* in the infection assays. In Figure 8(a), we show the viability of* S. aureus* from the supernatants obtained from the invasion assays (2 h).* S. aureus* employed in the invasion assays from the E2-treated bMEC cultures showed a reduced viability rate (60% of reduction) compared to the bacteria recovered from the supernatants of the vehicle-treated bMECs. This result suggests that antimicrobial components are present in the culture media from the bMECs treated with 50 pg/mL of E2. With the purpose of reinforcing these data, we incubated* S. aureus* for 2 h with 1 mL of conditioned media obtained from the vehicle- or E2-treated bMECs. According to Figure 8(b), the conditioned medium from the E2-treated bMECs reduces the number of CFU of* S. aureus* (a 60% of reduction). These results suggest that E2-treated bMECs secrete antimicrobial components. Next, we explored whether the gene expression of some antimicrobial elements was induced in the E2-treated bMECs.  Figure 8(c) shows the RT-qPCR analysis of different antimicrobial peptides, including several *β*-defensins and 1 psoriasin (S100A7). E2 upregulates the expression of the *β*-defensins bovine defensin *β*1 (DEFB1, ~5-fold) and Bovine Neutrophil Beta Defensin 5 (BNBD5, ~3-fold). In addition, E2 induces the expression of psoriasin S100A7 (~6.5-fold). In the presence of infection, only psoriasin S100A7 mRNA was induced (~13-fold). The other peptides analyzed remained unchanged in relation to the control cells. A similar behavior was observed in the cells treated with E2 and infected with* S. aureus*, where the expression of psoriasin was upregulated by ~9-fold. Considering these results, we measured the accumulation of DEFB1 in the bMEC medium by ELISA. In Figure 8(d), we show that E2 does not induce the secretion of DEFB1 in the culture medium, which is stimulated by infection.* S. aureus* infection in the E2-treated cells reduces the concentration of DEFB1 secreted in relation to infection alone. Thus, this is not the mechanism by which bacterial internalization is reduced in the E2-treated cells. In addition, the secretion of NO was not modified in the presence of E2 in the bMECs (Figure 8(e)), and thus it is not involved in the reduced internalization detected in the E2-treated bMECs.

## 4. Discussion

It is well known that, during the postpartum period, dairy cows experience an increased incidence of mammary infectious diseases, such as mastitis. This augmented frequency has been correlated with a decreased functionality of the innate immune system [3]. During this period, E2 levels rise abruptly in the last week before parturition, peak in the last 3 days before delivery, and fall rapidly after calving and regain basal values [3, 4]. It has been described that estrogens have both anti-inflammatory and proinflammatory functions, and their role in the modulation of the innate immune response of epithelial cells during infection has been documented [19]. It has been shown that E2 is required for mammary epithelial cell proliferation and ductal development in the growing animal [20]. In addition, the presence of ERs has been shown in this tissue [20]. The goal of this work was to study the immunomodulatory functions of E2 on bMECs during* S. aureus* infection, which have not been explored to date. According to previous reports showing immunomodulatory functions of E2 on bovine neutrophils, we evaluated E2 in a range of concentrations between 1 and 500 pg/mL [6]. Bacteria are capable of metabolizing sex steroid hormones, such as estradiol [21]. Thus, it is important to analyze whether E2 alters* S. aureus* viability or growth. At the incubation times and the range of E2 concentrations analyzed we did not detect a bacteriostatic or a bactericidal effect of E2 on* S. aureus* (Figure 1). In agreement, Hosoda et al. [22] showed that different steroid hormones were not bactericidal for* S. aureus*. In addition, at the range of concentrations tested, E2 was not toxic to the bovine mammary epithelial cells. Sobolewska et al. [23] have reported that similar concentrations of E2 and progesterone exert stimulatory effects on autophagy in bMECs after 48 h, which is implied during the involution of the bovine mammary gland in the dry period. In the present study, we cannot discard the possibility that longer incubation times with E2 could be cytotoxic for these cells.

In this work, we demonstrated that 50 pg/mL of E2 significantly inhibits ~50%* S. aureus* internalization into the bMECs (Figure 2). Because E2 does not have antibacterial activity, this reduction might be the result of the immunomodulatory effects that have been associated with this hormone. According to our results, the effect of E2 on* S. aureus* internalization shows a nonmonotonic dose-response curve. This behavior requires further investigation, however; nonmonotonic effects of E2 have been described on the development of mammary gland in* in vivo* models, as well as in human breast cancer cells. A possible explanation could be related to the versatility of E2 actions, which include the classic genomic functions, as well as nongenomic or membrane-initiated estrogen-signaling pathway [24, 25]. The inhibitory effect of E2 on* S. aureus* internalization cannot be attributable to the reduction in integrin *α*5*β*1 MA because this receptor does not participate in the internalization of* S. aureus* in E2-treated bMECs. In addition, although E2 reduces *α*5*β*1 MA, the presence of bacteria promotes its traffic at early times of infection (Figure 3), as we reported previously [10]. Considering this, the inhibition on* S. aureus* internalization in the E2-treated bMECs could be a consequence of the activation of immunomodulatory pathways. To explore this possibility, we analyzed the participation of the receptor TLR2 in this process. Accordingly, the results shown in Figure 4 suggest that E2 does not induce the MA of this receptor despite the fact that it upregulates the expression of its gene. Mammary epithelial cells play an essential role in the surveillance of mammary tissue during infections because they help in immune cell recruitment and bacterial recognition via the TLR signaling pathways [26, 27]. TLR2 is the major receptor for* S. aureus* detection and also mediates immunomodulatory functions [28]. We have previously demonstrated that* S. aureus* induces TLR2 MA and activation in bMECs in the same way that the lactogenic hormone prolactin stimulates* S. aureus* internalization in the bMECs [10]. E2 shows differential effects on TLR2 expression and/or activation; for example, in THP-1 cells, it increases the expression of TLR2 mRNA [29], but, in a bovine oviduct epithelial cell culture, it reduces the LPS-induced TLR2 mRNA expression [30]. Thus, the activation of the TLR2 pathways by E2 depends on the tissue and the physiological condition of the organism. In agreement with the absence of TLR2 activation in E2-treated bMECs, we detected that the MAPK p38 and ERK1/2 are inactivated in these cells (Figure 5). In addition, these kinases are not employed during the inhibition of* S. aureus* internalization induced by E2 (Figure 5). We have previously reported that these three MAPKs are required during* S. aureus* internalization into the bMECs [18].

We next explored whether E2 or infection regulates ERs activation because the direct effects of this hormone are exerted via intracellular ERs. In Figure 6, we showed that E2 induces the intracellular activation of ER*α*, as well as upregulating the expression of its gene. Likewise,* S. aureus* infection increases ER*α* mRNA expression in the E2-treated bMECs, but this induction is not related with ER*α* activation. In agreement, Yart et al. [31] reported that in a cell line of bMECs (MAC-T) the expression of ER*α* protein increases in response to E2 treatment. However, the activation of ER*α* by infection has not been extensively explored to date. Accordingly, Holm et al. [32] described that LPS from Gram-negative bacteria reduces ER*α* protein in endothelial cells. The presence of ER*β* in the nuclei from infected bMECs (Figure 6(c)) indicates the participation of this receptor during* S. aureus* infection, which has not been reported. Nevertheless, flow cytometry analysis reveals that E2 does not activate ER*β*. Taken together, these results suggest that E2 activates ER*α* in bMECs, which might be directly or indirectly implicated in the reduction of* S. aureus* internalization. Because we did not detect the activation of MAPKs in the E2-treated bMECs (Figure 5(c)) it is probable that E2 triggers the direct activation of ER*α* in bMECs, which up- and/or downregulates the transcription of various genes by binding to the estrogen response element of the genes or by interacting with other transcription factors [33]. To address this hypothesis, we analyzed some inflammatory genes in the E2-treated bMECs in the absence or presence of* S. aureus* infection. We previously reported that, with the exception of TNF-*α*,* S. aureus* inhibits the innate immune response of bMECs, as it is unable to induce the gene expression of some proinflammatory cytokines, such as IL-1*β* or IL-6 [10]. However, we also reported that, in the presence of ethanol as vehicle (2%),* S. aureus* slightly induces IL-1*β* mRNA expression [15]. The results from this work strengthen previous reports. Interestingly, E2 at 50 pg/mL significantly increases proinflammatory cytokine mRNA expression such as TNF-*α* and IL-1*β*, prior to* S. aureus* infection. However, upon infection, the levels of both cytokine mRNAs were downregulated in E2-treated bMECs. The differential influence of E2 on the expression of innate immune response genes depends on multiple factors, such as the hormone concentration or the physiological condition of the individuals [34]. These results coincide with other reports using urinary epithelial cells, where E2 reduces the LPS-induced cytokine expression [19]. Steroids have the ability to suppress (and/or resolve) an inflammatory response. In agreement with the anti-inflammatory function of E2 on bMECs, we detected a significant reduction in the secretion of proinflammatory cytokines (Figure 7(c)) in the E2-treated cells. This effect was maintained in the presence of* S. aureus*, with exception of IL-1*β*, which was induced in the infected E2-treated cells. This effect can be the consequence of the activation of other mechanisms, such as inflammasome activation, which requires further research [35]. Interestingly, and in agreement with the anti-inflammatory actions of the hormone, E2 also induces the mRNA expression of IL-10, an anti-inflammatory cytokine, which was upregulated by the hormone in the E2-treated cells challenged with* S. aureus*.

In addition to the immunomodulatory role of E2, it has been reported that this hormone also induces the production of antibacterial compounds in uterine epithelial cells [19]; a similar effect in the present model could explain the reduction in the internalization of* S. aureus*. Our data suggest that E2-treated bMECs produce an antibacterial compound, which is secreted into the culture medium (Figures 8(a) and 8(b)). To determine the origin of this activity, we analyzed the mRNA expression of different antimicrobial peptides (*β*-defensins and psoriasin). E2 induces the expression of DEFB1, BNBD5, and psoriasin S100A7 mRNAs. We also analyzed the secretion of DEFB1 into the culture medium, which was only induced by bacteria and not by E2. It is necessary to analyze the concentrations of BNBD5 or psoriasin in the culture medium to resolve if they are implicated in the reduction of* S. aureus* internalization in the E2-treated bMECs. Accordingly, Fahey et al. [19] determined that in uterine epithelial cells E2 exerts anti-inflammatory effects and enhances the production of antimicrobial peptides, which display direct defense actions during the infection of this tissue. It is possible that a similar effect could be occurring in bMECs, but further research is necessary to corroborate this possibility. In addition, the induction of psoriasin expression by E2 has been demonstrated in the human mammary epithelial cell line MCF-7 [36]. According to our results, E2 is not favoring the production of NO as a possible antimicrobial compound. Altogether, these results indicate that E2 at 50 pg/mL reduces* S. aureus* internalization in the bMECs by modulating the innate immune response through the activation of ER*α*. Because we did not detect ER*α* activation in* S. aureus*-challenged bMECs, we can hypothesize that 50 pg/mL of E2 during 24 h activates ER*α* in bMECs, which promotes a slightly anti-inflammatory response that is enhanced during infection. It is possible that during infection other mechanisms (i.e., transcription factors) are participating because the activation of ER*α* is turned off. In this process, the TLR2/MAPK pathway is not activated.

## 5. Conclusions

E2 (50 pg/mL) induces the anti-inflammatory response of the bMECs during* S. aureus* internalization through the participation of ER*α*. This leads to the increased production of antimicrobial molecules, favoring* S. aureus* elimination.

## Figures and Tables

**Table 1:** Oligonucleotides used in this study.

Gene (bovine)^*∗*^	Primer	Sequence (5′ → 3′)	Fragment size (bp)	Tm (°C)	References
TAP	F	GCGCTCCTCTTCCTGGTCCTG	216	57	[14]
	R	GCACGTTCTGACTGGGCATTGA			

LAP	F	GCCAGCATGAGGCTCCATC	194	54	[14]
	R	CTCCTGCAGCATTTTACTTGGGCT			

DEFB1	F	CCATCACCTGCTCCTCACA	185	54	[14]
	R	ACCTCCACCTGCAGCATT			

BNDB4	F	GCCAGCATGAGGCTCCATC	278	54	This study
	R	CGTTTAAATTTAGACGGTGT			

BNBD5	F	GCCAGCTGAGGCTCCATC	143	55	[14]
	R	TTGCCAGGGCACGAGATCG			

BNBD10	F	GCTCCATCACCTGCTCCTC	152	54	[14]
	R	AGGTGCCAATCTGTCTCATGA			

GAPDH	F	TCAACGGGAAGCTCACTGG	237	57	[14]
	R	CCCCAGCATCGAAGGTAGA			

*α*5 integrin	F	TGCAGTGTGAGGCCGTGTATGAAG	230	58	[10]
	R	CGGGAGGGAGCGTTTGAAGAAT			

*β*1 integrin	F	TGCGAGTGTGGTTGCCTGTAAGT	123	60	[10]
	R	ATTGAAGGCTCGGCACTGAACA			

TLR2	F	CGACTGGCCCGATGACTACC	146	58.5	[15]
	R	TGAGCAGGAGCAACAGGAAGAG			

TNF-*α*	F	CCCCTGGAGATAACCTCCCA	101	56	[15]
	R	CAGACGGGAGACAGGAGAGC			

IL-1*β*	F	GCAGAAGGGAAGGGAAGAATGTAG	198	52	[15]
	R	CAGGCTGGCTTTGAGTGAGTAGAA			

IL-6	F	AACCACTCCAGCCACAAACACT	179	57	[15]
	R	GAATGCCCAGGAACTACCACAA			

IL-8	F	TTCCACACCTTTCCACCCCAA	149	53.5	[15]
	R	GCACAACCTTCTGCACCCACTT			

IL-10	F	GATGCGAGCACCCTGTCTGA	129	59	[15]
	R	GCTGTGCAGTTGGTCCTTCATT			

S100A7 (psoriasin)	F	GCAGCTCTCAGCTTGAGCAG	221	54	[14]
	R	CCAGCAAGGACAGGAACTCAG			

ER*α*	F	TTCGGAAGTGCTATGAGGTTG	139	55	This study
	R	GATATTCTTTGTGTTGGAGTTTTA			

ER*β*	F	GACCCTACCAGACCTTTCAGTG	153	60	This study
	R	TGCTCCATGCCTTTGTTGC			

^*∗*^TAP: Tracheal Antimicrobial Peptide; LAP: Lingual Antimicrobial Peptide; DEFB1: Defensin Beta 1; BNBD4: Bovine Neutrophil Beta Defensin 4; BNBD5: Bovine Neutrophil Beta Defensin 5; BNDB10: Bovine Neutrophil Beta Defensin 10; GAPDH: Glyceraldehyde 3-phosphate dehydrogenase; TLR2: Toll-Like Receptor 2; TNF-*α*: Tumor Necrosis Factor-*α*; IL-1*β*: Interleukin 1-*β*; IL-6: Interleukin-6; IL-8: Interleukin-8; IL-10: Interleukin-10; S100A7: S100 Calcium-binding protein A7; ER*α*: Estrogen Receptor *α*; ER*β*: Estrogen Receptor *β*.
